# Exploring cocoa bean fermentation mechanisms by kinetic modelling

**DOI:** 10.1098/rsos.210274

**Published:** 2022-02-16

**Authors:** Mauricio Moreno-Zambrano, Matthias S. Ullrich, Marc-Thorsten Hütt

**Affiliations:** Department of Life Sciences and Chemistry, Jacobs University Bremen, Campus Ring 1, 28759 Bremen, Germany

**Keywords:** theoretical biology, Bayesian parameter estimation, cocoa bean fermentation, kinetic modelling

## Abstract

Compared with other fermentation processes in food industry, cocoa bean fermentation is uncontrolled and not standardized. A detailed mechanistic understanding can therefore be relevant for cocoa bean quality control. Starting from an existing mathematical model of cocoa bean fermentation we analyse five additional biochemical mechanisms derived from the literature. These mechanisms, when added to the baseline model either in isolation or in combination, were evaluated in terms of their capacity to describe experimental data. In total, we evaluated 32 model variants on 23 fermentation datasets. We interpret the results from two perspectives: (1) success of the potential mechanism, (2) discrimination of fermentation protocols based on estimated parameters. The former provides insight in the fermentation process itself. The latter opens an avenue towards reverse-engineering empirical conditions from model parameters. We find support for two mechanisms debated in the literature: consumption of fructose by lactic acid bacteria and production of acetic acid by yeast. Furthermore, we provide evidence that model parameters are sensitive to differences in the cultivar, temperature control and usage of steel tanks compared with wooden boxes. Our results show that mathematical modelling can provide an alternative to standard chemical fingerprinting in the interpretation of fermentation data.

## 1. Introduction

Cocoa beans from *Theobroma cacao* L. are the raw material of chocolate. Their fermentation plays a fundamental role as being responsible for eliminating undesired properties from freshly harvested beans, e.g. astringency and bitterness, besides yielding chocolate-related flavour and aroma precursor compounds [1,2]. In contrast to the highly controlled fermentation processes known from other food products, this process is conducted *in situ* at each of the producing farms in a spontaneous form varying in both, methodology, e.g. wooden boxes, heaps and platforms [2–4], and observed microbial diversity [5].

This heterogeneity due to different fermentation methods and indigenous microbiota, leads to a plethora of studies that have qualitatively described the process, e.g. [4,5]. Among all these, sequentiality of microbial populations thriving on the beans’ enclosing pulp constitutes the process dynamics with greatest acceptance [1,2,4].

In further detail, regardless of the wide range of factors that could differentiate fermentation trials, sequential succession of microbial groups during their execution can be understood as a three-phased process, where a microbial group dominates each phase during a distinct time period. In a first stage, anaerobic conditions due to the packed nature of the pulp favour the growth of yeasts that bloom as a consequence of a carbohydrate-rich environment producing mainly ethanol. Through their pectinolytic action yeasts drive to a liquefaction of the pulp. As a consequence, a drainage of pulp permits air to enter into the fermenting mass, contributing to the decline of yeast population. Under these conditions, a second stage is dominated by the growth of microaerophilic lactic acid bacteria (LAB) that at the onset of the process were reproducing at a lower rate than yeasts. Therefore, by depletion of remaining sugars from the first stage, LAB yield mainly lactic and acetic acids. At this point, after considerable drainage of pulp, a fully aerobic phase is reached. This third and final stage is characterized by an almost complete dominance of aerophilic acetic acid bacteria (AAB) that oxidize lactic acid into acetoin, and ethanol into acetic acid [1,3,4,6].

As a consequence, microbial sequentiality during the fermentation has served as the basis in formulating a few mathematical approaches for its quantitative description [7–10]. Among these, we previously proposed a mathematical model of ordinary differential equations (ODEs), which served us as baseline here [9]. The main sub-processes implemented in this study focus on the activity of major microbial groups, namely yeasts (Y), LAB and AAB. As a result, we developed a successful model based on well-known regulatory assumptions: in a first instance, Y come into play by converting glucose (Glc) and fructose (Fru) into ethanol (EtOH). Concomitantly, LAB consumes Glc leaving as products lactic acid (LA) and acetic acid (Ac). Finally, AAB takes over the last phase of fermentation by oxidizing EtOH and LA into Ac [9].

Beyond these main components, more regulatory mechanisms have been mentioned across experimental studies that could bring more insight into the dynamics of cocoa bean fermentation. Among these, we here put special emphasis in five phenomena (see detailed references in Materials and methods, below): (i) decrease of product metabolites by physical causes, (ii) consumption of Fru by LAB, (iii) production of Ac by Y, (iv) consumption of LA by Y, and (v) over-oxidation of Ac by AAB.

Along these lines, we were able to assess the plausibility of stand-alone and simultaneous occurrence of these mechanisms when added to our baseline model and to identify systematic differences of fermentation features by applying classification methods over their resulting vectors of parameter estimates. Our key questions are: (i) Which model variants describe the experimental data better than the baseline model? (ii) For which model can parameter differences be related to differences in the fermentation process?

## 2. Material and methods

### 2.1. Identification and processing of experimental data

A literature survey concerning cocoa bean fermentation trials was performed with the purpose of gathering experimental data. Reported trials considered in this study were papers published between 2000 and 2019. As inclusion criteria, only English-written works with time series of minimum five observations for metabolites Glc, Fru, EtOH, LA and Ac, besides total population counts of Y, LAB and AAB were included.

In all cases, population growth of Y, LAB and AAB were transformed from log base 10 of colony forming units (log_10_(CFU)) to milligrams of microbial group (MG) per gram of pulp (mg(MG)g(pulp)^−1^) as these are the units in which most kinetic single-strained microbial growth studies report their dynamics as well as their dependent constants, i.e. specific maximum growth and mortality rates, and yield coefficients. Moreover, with the purpose of facilitating the estimation of models’ parameters by avoiding numerical issues during their calibration, all time series were scaled by dividing each observation by its own maximum value. Hence, obtained parameter estimates were rescaled to their original units by using simple transformations for their further comparison with previously reported values for single-strained microbial studies (see electronic supplementary material, S1) [9].

For this current research, distinct trials were given a code name based on country of origin and fermentation method. A complete detail of data included in this research where at least one model variation successfully fit it (see following sections for their explanation) is shown in table 1. For a comprehensive list of all data initially considered see electronic supplementary material, S2.

**Table 1.** Considered data sources.

reference	year	country	cultivar	method	trial	code	turning	Ctrl. Temp.
Camu *et al.* [11]	2007	Ghana	Criollo/Forastero	heap	heap 5	ghhp1	✗	✗
Lagunes Gálvez *et al.* [12]	2007	Dominican Republic	Trinitario	wooden box	NA	dowb1	✓	✗
Camu *et al.* [13]	2008	Ghana	NA*	heap	heap 10	ghhp2	✓	✗
					heap 11	ghhp3	✗	✗
					heap 12	ghhp4	✓	✗
					heap 13	ghhp5	✗	✗
Papalexandratou *et al.* [14]	2011	Brazil	Criollo/Forastero	wooden box	box 1	brwb1	✓	✗
					box 2	brwb2	✓	✗
Papalexandratou *et al.* [15]	2011	Ecuador	Nacional/Trinitario	platform	P1	ecpt1	✗	✗
					P2	ecpt2	✗	✗
				wooden box	B1	ecwb1	✓	✗
					B2	ecwb2	✓	✗
Pereira *et al.* [16]	2012	Brazil	NA*	plastic box	PC	brpb1	✓	✓
				stainless tank	ST	brst1	✓	✓
Pereira *et al.* [17]	2013	Brazil	Mixed hybrids*	wooden box	WB1	brwb3	✓	✗
					WB2	brwb4	✓	✗
				stainless tank	SST	brst2	✓	✗
Moreira *et al.* [18]	2013	Brazil	PH16	wooden box	PH16	brwb7	NA	✗
Papalexandratou *et al.* [19]	2013	Malaysia	Mixed hybrids	wooden box	box 2	mywb3	✓	✗
Romanens *et al.* [20]	2018	Honduras	IMC-67, UF-29, UF-668	wooden box	OF-F	hnwb1	✓	✗
Lee *et al.* [21]^†^	2019	Ecuador	Criollo	plastic box	NA	ecpb1	NA	✓
Papalexandratou *et al.* [22]	2019	Nicaragua	Nugu/O’payo	wooden box	NUGU	niwb1	✓	✗
					O’PAYO	niwb2	✓	✗

Only fermentation trials that were successfully described by at least one model iteration (MI) are listed. Author, year of publication, cocoa country of origin, cocoa cultivar, used methodology, code name given in the original trial, recoded given name in this research, turning of the fermenting mass and controlled temperature are shown.

*Unidentified cultivars used by Camu *et al.* [13], Pereira *et al.* [16] and Pereira *et al.* [17] were coded as *un1*, *un2* and *un3*, respectively, for further PCA.

^†^Simulated fermentation.

### 2.2. Formulation of candidate models

Starting from the baseline model from [9], we implemented five regulatory mechanisms that have been reported or hypothesized in multiple studies. In the following paragraphs, the baseline model will be described and proposed mechanisms reasoning will be presented conforming what we considered their likeliness of occurrence as (i) decay of fermentation’s products, (ii) consumption of Fru by LAB, (iii) production of Ac by Y, (iv) consumption of LA by Y, and (v) over-oxidation of Ac by AAB.

#### 2.2.1. Baseline model

**Figure 1.**
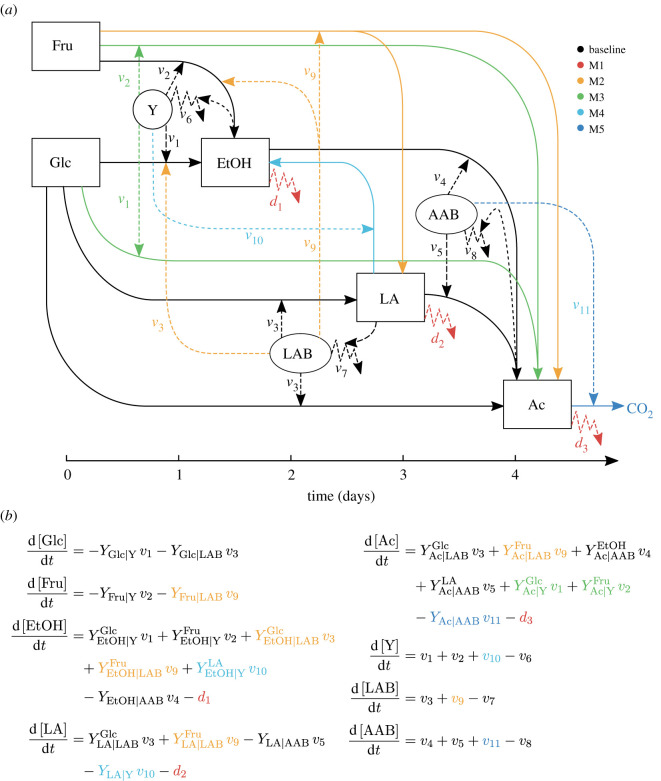
Summary of models iterations. (*a*) Network diagram of mechanisms over baseline model. Microbial groups: yeast (Y), lactic acid bacteria (LAB) and acetic acid bacteria (AAB) are represented as circles. Metabolites: glucose (Glc), fructose (Fru), ethanol (EtOH), lactic acid (LA) and acetic acid (Ac) are represented as squares. The growth rates of Y on Glc (*v*_1_), Fru (*v*_2_) and LA (*v*_10_), of LAB on Glc (*v*_3_) and Fru (*v*_9_), and of AAB on EtOH (*v*_4_), LA (*v*_5_) and Ac (*v*_11_) are represented as straight dashed arrows. The mortality rates of Y (*v*_6_), LAB (*v*_7_) and AAB (*v*_8_) are represented as zigzag dashed arrows as the decay rates of EtOH (*d*_1_), LA (*d*_2_) and Ac (*d*_3_). Straight dashed arrows pointing from products to mortality rates represent product influence on mortality rates. Solid straight arrows show the direction in which the conversion of metabolites occur. Baseline model by Moreno-Zambrano *et al.* [9] comprehends mechanisms depicted in black (circle). (*b*) Representation of full model with mechanisms M1, M2, M3, M4 and M5 together. M1 (red circle), encompasses losses of EtOH, LA and Ac. M2 (orange circle), involves conversion of Glc into EtOH, and Fru into EtOH, LA and Ac by LAB. M3 (green circle), comprises conversion of Glc and Fru into Ac by Y. M4 (light blue circle), refers to conversion of LA into EtOH by Y. M5 (dark blue circle), represents over-oxidation of Ac by AAB.

As baseline, we used our previously developed model [9] that consist of eight ODEs describing the dynamics of metabolites: Glc, Fru, EtOH, LA and Ac, besides microbial groups: Y, LAB and AAB. Both metabolites and microbial groups are interdependent in the dynamic process by means of growth and mortality rates of the latter (figure 1*a*). Monod [23] and Contois [24] type equations were employed to describe the growth rates of microbial groups. Growth rates *v*_1_ and *v*_2_ of Y on Glc and Fru, respectively, as well as growth rates *v*_3_ of LAB on Glc and *v*_4_ of AAB on EtOH, correspond to Monod equations, while the growth of AAB on LA, *v*_5_, corresponds to a Contois term. Mortality rates of Y, LAB and AAB were modelled as Chick–Watson equations [25] by considering second- and third-order death kinetics, as shown in table 2.

**Table 2.** Growth, mortality and decay rates for cocoa bean fermentation models.

growth rate equation	mortality rate equation	decay rate equation
$v_{1}=\frac{\mu_{\max}^{Y_{\mathrm{Glc}}}[\mathrm{Glc}]}{[\mathrm{Glc}]+K_{\mathrm{Glc}}^{Y}}[Y]$		
$v_{2}=\frac{\mu_{\max}^{Y_{\mathrm{Fru}}}[\mathrm{Fru}]}{[\mathrm{Fru}]+K_{\mathrm{Fru}}^{Y}}[Y]$	*v*_6_ = *k*_Y_ [Y] [EtOH]	*d*_1_ = *b*_EtOH_ [EtOH]
$v_{10}=\frac{\mu_{\max}^{Y_{\mathrm{LA}}}[\mathrm{LA}]}{[\mathrm{LA}]+K_{\mathrm{LA}}^{Y}}[Y]$		
$v_{3}=\frac{\mu_{\max}^{{\mathrm{LAB}}_{\mathrm{Glc}}}[\mathrm{Glc}]}{[\mathrm{Glc}]+K_{\mathrm{Glc}}^{\mathrm{LAB}}}[\mathrm{LAB}]$	*v*_7_ = *k*_LAB_ [LAB] [LA]	*d*_2_ = *b*_LA_ [LA]
$v_{9}=\frac{\mu_{\max}^{{\mathrm{LAB}}_{\mathrm{Fru}}}[\mathrm{Fru}]}{[\mathrm{Fru}]+K_{\mathrm{Fru}}^{\mathrm{LAB}}}[\mathrm{LAB}]$		
$v_{4}=\frac{\mu_{\max}^{{\mathrm{AAB}}_{\mathrm{EtOH}}}[\mathrm{EtOH}]}{[\mathrm{EtOH}]+K_{\mathrm{EtOH}}^{\mathrm{AAB}}}[\mathrm{AAB}]$		
$v_{5}=\frac{\mu_{\max}^{{\mathrm{AAB}}_{\mathrm{LA}}}[\mathrm{LA}]}{\left[\mathrm{LA}]+K_{\mathrm{LA}}^{\mathrm{AAB}}[\mathrm{AAB}\right]}[\mathrm{AAB}]$	*v*_8_ = *k*_AAB_ [AAB] [Ac]^2^	*d*_3_ = *b*_Ac_ [Ac]
$v_{11}=\frac{\mu_{\max}^{{\mathrm{AAB}}_{\mathrm{Ac}}}[\mathrm{Ac}]}{[\mathrm{Ac}]+K_{\mathrm{Ac}}^{\mathrm{AAB}}}[\mathrm{AAB}]$		

Microbial groups: yeast (Y), lactic acid bacteria (LAB) and acetic acid bacteria (AAB). Metabolites: glucose (Glc), fructose (Fru), ethanol (EtOH), lactic acid (LA) and acetic acid (Ac). Microbial groups and metabolites are expressed as concentrations, both within square brackets [ ]. Maximum specific growth rates $\mu_{\max}^{i_{n}}$, correspond to the maximum growth rate of microbial group *i*, growing on substrate *n*. Substrate saturation constants $K_{m}^{i}$, correspond to the substrate saturation constant of microbial group *i*, growing on substrate *m*. Constant mortality rates *k*_*i*_, correspond to mortality of microbial group *i*. Decay rates *d*_*j*_, correspond to decay rate of metabolite *j*. All rates with the exception of *d*_1_, *d*_2_, *d*_3_, *v*_9_, *v*_10_ and *v*_11_, are part of the baseline model as proposed by Moreno-Zambrano *et al.* [9].

The model contains 24 parameters: five maximum specific growth rates, five substrate saturation constants, three mortality rate constants and 11 yield coefficients as depicted in table 2 and the following equations:

2.1 $$\frac{d[\mathrm{Glc}]}{dt}=-Y_{\mathrm{Glc}|Y}\;v_{1}-Y_{\mathrm{Glc}|\mathrm{LAB}}\;v_{3}$$

2.2 $$\frac{d[\mathrm{Fru}]}{dt}=-Y_{\mathrm{Fru}|Y}\;v_{2}$$

2.3 $$\frac{d[\mathrm{EtOH}]}{dt}=Y_{\mathrm{EtOH}|Y}^{\mathrm{Glc}}\;v_{1}+Y_{\mathrm{EtOH}|Y}^{\mathrm{Fru}}\;v_{2}-Y_{\mathrm{EtOH}|\mathrm{AAB}}\;v_{4}$$

2.4 $$\frac{d[\mathrm{LA}]}{dt}=Y_{\mathrm{LA}|\mathrm{LAB}}^{\mathrm{Glc}}\;v_{3}-Y_{\mathrm{LA}|\mathrm{AAB}}\;v_{5}$$

2.5 $$\frac{d[\mathrm{Ac}]}{dt}=Y_{\mathrm{Ac}|\mathrm{LAB}}^{\mathrm{Glc}}\;v_{3}+Y_{\mathrm{Ac}|\mathrm{AAB}}^{\mathrm{EtOH}}\;v_{4}+Y_{\mathrm{Ac}|\mathrm{AAB}}^{\mathrm{LA}}\;v_{5}$$

2.6 $$\frac{d[Y]}{dt}=v_{1}+v_{2}-v_{6}$$

2.7 $$\frac{d[\mathrm{LAB}]}{dt}=v_{3}-v_{7}$$

and

2.8 $$\frac{d[\mathrm{AAB}]}{dt}=v_{4}+v_{5}-v_{8}.$$

In regard to our proposed mechanisms, their inclusion into the baseline model is conducted by adding extra growth and mortality rates, as well as linear terms when needed (table 2). For a deeper look into their mathematical formulation, see electronic supplementary material, S3.

#### 2.2.2. Mechanism 1: decay of fermentation products

Mechanism 1 (M1) is based on concentration decline of product metabolites at later stages of fermentation that has been hypothesized as a consequence of both physical and biological constraints. Here, we will take into account the first group only. Among these, volatile compounds (e.g. EtOH and Ac) might decrease as a result of evaporation and leakage of fermentation sweating [3,6,11,13]. Regarding non-volatile compounds (e.g. LA), the widely described diffusion process of metabolites from the pulp into the cocoa bean, might also play an important role in their reduction [4,6,26].

#### 2.2.3. Mechanism 2: consumption of Fru by LAB

Opposed to our original approach of modelling LAB growth exclusively based on Glc uptake [9], mechanism 2 (M2) takes into account obligatory and facultatively heterofermentative species which are capable of using Glc and Fru as carbon sources (e.g. *Limosilactobacillus fermentum* and *Lactiplantibacillus plantarum*, respectively) with an accompanying production of EtOH besides LA and Ac [3,4,12,22,27,28].

#### 2.2.4. Mechanism 3: production of Ac by Y

Mechanism 3 (M3) is based on the evidence of among fermentation products that Y generate (e.g. ethanol, glycerol and carbon dioxide), Ac can be created through pyruvate metabolism and tricarboxylic acid cycle [3,4,16]. Besides, under controlled conditions, production of Ac by Y could explain concentrations of Ac that do not correspond to AAB’s population sizes [17].

#### 2.2.5. Mechanism 4: consumption of LA by Y

In mechanism 4 (MF4), during the first stage of fermentation, the Y population prevails due to the anaerobic conditions in the pulp. However, under an aerobic environment as during the third stage, yeasts such as *S. cerevisiae* are capable to oxidize LA to produce pyruvate [29,30]. Additionally, other species of yeast (e.g. *Pichia fermentans* and *Candida krusei*) can assimilate LA and produce EtOH.

#### 2.2.6. Mechanism 5: over-oxidation of Ac by AAB

In mechanism 5 (M5), during the final stage of fermentation, AAB dominates microbial population by taking advantage of a fully aerobic environment, while consuming EtOH and LA previously produced by Y and LAB, respectively. Once EtOH has mostly diminished, it has been argued that AAB starts over-oxidizing Ac into carbon dioxide, which would lead to halting the cocoa fermentation process due to an increase of temperature that results in the declining of Y, LAB and AAB [3,4,6,31].

A comprehensive graphical representation of all proposed mechanisms is shown in figure 1*a*, full model including all mechanisms here proposed is shown in figure 1*b* and a detailed interpretation of all model parameters is shown in table 3.

**Table 3.** Parameters of the cocoa bean fermentation baseline model and proposed mechanisms.

parameter	mechanism	units	interpretation
$\mu_{\max}^{Y_{\mathrm{Glc}}}$	B	h^−1^	maximum specific growth rate of Y on Glc
$\mu_{\max}^{Y_{\mathrm{Fru}}}$	B	h^−1^	maximum specific growth rate of Y on Fru
$\mu_{\max}^{Y_{\mathrm{LA}}}$	M4	h^−1^	maximum specific growth rate of Y on LA
$\mu_{\max}^{{\mathrm{LAB}}_{\mathrm{Glc}}}$	B	h^−1^	maximum specific growth rate of LAB on Glc
$\mu_{\max}^{{\mathrm{LAB}}_{\mathrm{Fru}}}$	M2	h^−1^	maximum specific growth rate of LAB on Fru
$\mu_{\max}^{{\mathrm{AAB}}_{\mathrm{EtOH}}}$	B	h^−1^	maximum specific growth rate of AAB on EtOH
$\mu_{\max}^{{\mathrm{AAB}}_{\mathrm{LA}}}$	B	h^−1^	maximum specific growth rate of AAB on LA
$\mu_{\max}^{{\mathrm{AAB}}_{\mathrm{Ac}}}$	M5	h^−1^	maximum specific growth rate of AAB on Ac
$K_{\mathrm{Glc}}^{Y}$	B	mg(Glc)g(pulp)^−1^	substrate saturation constant of Y growth on Glc
$K_{\mathrm{Fru}}^{Y}$	B	mg(Fru)g(pulp)^−1^	substrate saturation constant of Y growth on Fru
$K_{\mathrm{LA}}^{Y}$	M4	mg(Fru)g(pulp)^−1^	substrate saturation constant of Y growth on LA
$K_{\mathrm{Glc}}^{\mathrm{LAB}}$	B	mg(Glc)g(pulp)^−1^	substrate saturation constant of LAB growth on Glc
$K_{\mathrm{Fru}}^{\mathrm{LAB}}$	M2	mg(Fru)g(pulp)^−1^	substrate saturation constant of LAB growth on Fru
$K_{\mathrm{EtOH}}^{\mathrm{AAB}}$	B	mg(EtOH)g(pulp)^−1^	substrate saturation constant of AAB growth on EtOH
$K_{\mathrm{LA}}^{\mathrm{AAB}}$	B	mg(LA)g(pulp)^−1^	substrate saturation constant of AAB growth on LA
$K_{\mathrm{Ac}}^{\mathrm{AAB}}$	M5	mg(Ac)g(pulp)^−1^	substrate saturation constant of AAB growth on Ac
*k*_Y_	B	mg(EtOH)^−1^h^−1^	mortality rate constant of Y
*k*_LAB_	B	mg(LA)^−1^h^−1^	mortality rate constant of LAB
*k*_AAB_	B	mg(Ac)^−2^h^−1^	mortality rate constant of AAB
*Y*_Glc|Y_	B	mg(Glc)mg(Y)^−1^	Y-to-Glc yield coefficient
*Y*_Glc|LAB_	B	mg(Glc)mg(LAB)^−1^	LAB-to-Glc yield coefficient
*Y*_Fru|Y_	B	mg(Fru)mg(Y)^−1^	Y-to-Fru yield coefficient
*Y*_Fru|LAB_	M2	mg(Fru)mg(LAB)^−1^	LAB-to-Fru yield coefficient
$Y_{\mathrm{EtOH}|Y}^{\mathrm{Glc}}$	B	mg(EtOH)mg(Y)^−1^	Y-to-EtOH from Glc yield coefficient
$Y_{\mathrm{EtOH}|Y}^{\mathrm{Fru}}$	B	mg(EtOH)mg(Y)^−1^	Y-to-EtOH from Fru yield coefficient
$Y_{\mathrm{EtOH}|Y}^{\mathrm{LA}}$	M4	mg(EtOH)mg(Y)^−1^	Y-to-EtOH from LA yield coefficient
$Y_{\mathrm{EtOH}|\mathrm{LAB}}^{\mathrm{Glc}}$	M2	mg(EtOH)mg(LAB)^−1^	LAB-to-EtOH from Glc yield coefficient
$Y_{\mathrm{EtOH}|\mathrm{LAB}}^{\mathrm{Fru}}$	M2	mg(EtOH)mg(LAB)^−1^	LAB-to-EtOH from Fru yield coefficient
*Y*_EtOH|AAB_	B	mg(EtOH)mg(AAB)^−1^	AAB-to-EtOH yield coefficient
$Y_{\mathrm{LA}|\mathrm{LAB}}^{\mathrm{Glc}}$	B	mg(LA)mg(LAB)^−1^	LAB-to-LA from Glc yield coefficient
$Y_{\mathrm{LA}|\mathrm{LAB}}^{\mathrm{Fru}}$	M2	mg(LA)mg(LAB)^−1^	LAB-to-LA from Fru yield coefficient
*Y*_LA|AAB_	B	mg(LA)mg(AAB)^−1^	AAB-to-LA yield coefficient
*Y*_LA|Y_	M4	mg(LA)mg(Y)^−1^	Y-to-LA yield coefficient
$Y_{\mathrm{Ac}|\mathrm{LAB}}^{\mathrm{Glc}}$	B	mg(Ac)mg(LAB)^−1^	LAB-to-Ac from Glc yield coefficient
$Y_{\mathrm{Ac}|\mathrm{LAB}}^{\mathrm{Fru}}$	M2	mg(Ac)mg(LAB)^−1^	LAB-to-Ac from Fru yield coefficient
$Y_{\mathrm{Ac}|\mathrm{AAB}}^{\mathrm{EtOH}}$	B	mg(Ac)mg(AAB)^−1^	AAB-to-Ac from EtOH yield coefficient
$Y_{\mathrm{Ac}|\mathrm{AAB}}^{\mathrm{LA}}$	B	mg(Ac)mg(AAB)^−1^	AAB-to-Ac from LA yield coefficient
$Y_{\mathrm{Ac}|Y}^{\mathrm{Glc}}$	M3	mg(Ac)mg(Y)^−1^	Y-to-Ac from Glc yield coefficient
$Y_{\mathrm{Ac}|Y}^{\mathrm{Fru}}$	M3	mg(Ac)mg(Y)^−1^	Y-to-Ac from Fru yield coefficient
*Y*_Ac|AAB_	M5	mg(Ac)mg(AAB)^−1^	AAB-to-Ac yield coefficient
*b*_EtOH_	M1	h^−1^	decay rate of EtOH
*b*_LA_	M1	h^−1^	decay rate of LA
*b*_Ac_	M1	h^−1^	decay rate of Ac

Microbial groups: yeast (Y), lactic acid bacteria (LAB) and acetic acid bacteria (AAB). Metabolites: glucose (Glc), fructose (Fru), ethanol (EtOH), lactic acid (LA) and acetic acid (Ac). B, M1, M2, M3, M4 and M5 refer to baseline model and mechanisms 1 to 5, respectively.

### 2.3. Models iterations

For the purpose of checking the plausibility of different mechanisms working together, a series of model variants with combinations of M1, M2, M3, M4 and M5 were created, starting from the baseline model. Hence, 31 MIs plus the baseline model were object of being fitted to experimental data under a Bayesian parameter estimation framework. Each MI is labelled according to the mechanisms involved. For example, the full model containing all five proposed regulatory schemes is labelled MI(1,2,3,4,5), while the baseline is labelled MI(0).

### 2.4. Kinetic parameter estimation

The number of parameters among MIs constructed over combination of mechanisms ranges from 24 in the baseline model, to 43 in the full model including all mechanisms. In each case, a general Bayesian framework was used to sample their posterior distributions, where means were taken as point estimates with their corresponding 95% credible intervals (CIs) [9].

#### 2.4.1. Bayesian framework

First, let us consider any of our proposed deterministic MIs represented in a general form

2.9 $$\frac{dx_{i}}{dt}=f\;(x,\theta ),$$

where *x* represents a vector of state variables, *x*_*i*_ is its *i*th component and the function *f*(*x*, *θ*) summarizes the dependence of the right-hand side of the ODEs on *x* and all *k* model parameters [*θ*_1_, *θ*_2_, …, *θ*_*k*_] contained in vector *θ*.

If we assume that parameters *θ* are selected such that a set of data Y is described, a way to infer them is to compute the (posterior) probability of *θ* given Y, P(θ∣Y), which by applying Bayes’ theorem, is equal to

2.10 $$P(\theta |Y)=\frac{P(Y|\theta )\;P(\theta )}{P(Y)}.$$

Here, since P(Y) is a normalizing constant allowing the posterior density to integrate to one, equation (2.10) can be written in terms of the likelihood of observing Y given *θ*, P(Y∣θ), and the prior distribution of vector *θ*, *P*(*θ*), as

2.11 P(θ∣Y)∝P(Y∣θ) P(θ).

If we take into account that each component of Y contains *T* time steps, with *N* state variables being observed, equation (2.11) takes the form of a product over all series and each of their measured points as

2.12 $$P(\theta |Y)\propto \prod_{i=1}^{N}\prod_{\;j=1}^{T}P(Y_{i,j}|\theta )\;P(\theta ).$$

Finally, as our purpose is to identify values of *θ* that lead to a best agreement between $Y_{i,j}$ in equation (2.12) and *x*_*i*_(*j*) in equation (2.9), we can consider $Y_{i,j}$ to be sampled from a normal distribution whose mean is equal to the model’s prediction *f*(*x*, *θ*), with a standard deviation term, *σ*, (caused by noise of any kind) allowing us to reformulate the total posterior distribution as

2.13 $$P(\theta |Y)\propto \prod_{i=1}^{N}\prod_{\;j=1}^{T}N(f\;(x_{i,j},\theta ),\sigma )\;P(\theta ).$$

Hence, by applying the total posterior distribution in equation (2.13), an extra parameter corresponding to a total standard deviation, *σ*, is also estimated.

#### 2.4.2. Choice of priors

The regularization procedure of the data described in §2.1 permits to reduce the parameter search space in a convenient way for choosing the priors in ranges between 0 and approximately 1, which brings three main advantages: (i) independent prior distributions for each parameter can take the same form, (ii) by introducing scale information of the original units in which the parameters of the models are originally measured, we can formulate weakly informative priors capable of covering all possible values in the scaled space [9,32], and (iii) by avoiding diffuse priors, further model comparisons will be less likely to be affected by common problems, such as over-fitting [33] and ill-defined posteriors [34].

Hence, posterior distributions of *θ* and *σ* were computed using a normal distribution with mean 0.5 and standard deviation of 0.3 for each element of the parameter vector *θ* and a Cauchy distribution with location 0 and scale of 1 for *σ*. With the purpose of avoiding estimates with negative values, both priors were truncated to the positive set of real numbers,

2.14 $$\begin{array}{cc} & \theta_{k}∼N(0.5,\;0.3),\;\theta_{k}>0\\ \;\mathrm{and}\; & \sigma ∼C(0,\;1),\;\;\;\sigma >0. \end{array}\}$$

For a detailed description of prior distributions rescaled to the parameters’ original units see electronic supplementary material, table S2.

#### 2.4.3. Implementation

The fit of MIs to experimental data was performed with Stan [35] via RStan package in R [36,37]. Posterior distributions of *θ*, *σ* and *f*(*x*_*i*,*j*_, *θ*), were obtained by Markov chain Monte Carlo (MCMC) no-U-turn sampler (NUTS) method [38]. Each model was treated as an initial value problem, where ODEs were solved by the built-in Stan numerical solver *rk45* for non-stiff systems by means of fourth- and fifth-order Runge–Kutta method [39,40] with relative and absolute tolerance values of 1 × 10^−6^ for both, and a maximum number of steps of 1 × 10^4^. All MIs were fitted to data by running four parallel Markov chains of 3000 iterations each, with 1000 of them used for warm-up. Sampling convergence was assessed by examining $\hat{R}$ statistic, bulk effective sample size (bulk-ESS) and tail effective sample size (tail-ESS) as described by Vehtari *et al.* [41]. In cases where either bulk-ESS or tail-ESS were rejected at first, calibration routine was rerun doubling iterations (2000 for warm-up, 6000 in total) before reporting non-convergence. Assessing autocorrelation of the sampled parameters was performed by means of averaging computed effective sample sizes over number of posterior draws (ESS/N_draws_) and checking whether these were above 12.5%, meaning that as minimum 1000 ESS were obtained [42] (a more detailed explanation on the convergence criteria is described in electronic supplementary material, S4).

### 2.5. Model assessment

Under the umbrella of the proposed Bayesian framework, it is important to define what will be called from now on a *successful fit*. For this aspect, we consider as such, any MI fit that converged according to the criteria described in §2.4.3 and does not involve any reparametrization or use of distinct priors in cases were divergences of MCMC-NUTS or complete lack of sampling could arise as a result of complicated geometries imposed on the posterior distributions by inclusion of the assessed mechanisms over particular datasets. With this in mind, the quality of the models was assessed from two perspectives: (i) success of each MI across all data and (ii) predictive accuracy comparison of all MIs for each dataset.

On the one hand, Bayesian model averaging (BMA [43]) weights were computed using pseudo-BMA [44]. These weights, understood as representations of the relative probability of each MI [45], were then averaged over the total number of datasets that were fitted at least once by any MI, and used as a measure of the adequacy of each MI across all data. For computing the mean value of BMA weights (BMA_*w*_), non-successful fits were assigned values of zero. Furthermore, an observed success rate (OSR) and expected success rate (ESR) were determined on the basis of times where the model was satisfactorily fit to a given dataset. OSR is then defined by the ratio of the number of successful fits and the total of datasets fitted by at least one MI. ESR, used to properly compare the success rates of models with only a single additional mechanism with those models containing a combination of mechanisms, is defined as the product of the OSRs of the elementary MIs. For model variant MI(1,2,5), for example, the expected success rate is then the product of the observed success rates of the elementary models MI(1), MI(2) and MI(5).

On the other hand, all MIs that were suitably fitted to each dataset were compared by means of Pareto-smoothed importance sampling leave-one-out cross validation (PSIS-LOO, [46]) with the aim of checking whether a certain MI could perform outstandingly better than its counterparts in terms of its predictive accuracy.

### 2.6. Principal components analysis

In an approach of linking our mathematical exploration of mechanisms with a real-life application (where principal component analysis (PCA) is a common approach), we studied whether projecting the obtained vectors of kinetic parameters on the PCA space allows for a separation (and hence classification) of experiments according to fermentation features (e.g. cocoa beans’ country of origin and used cultivar), which are of interest in both academic research and the chocolate industry.

Consequently, six main features of fermentation trials were taken into account as groups and analysed via PCA over parameter estimates for all successful MIs. Of these, three consist of multiple classes: (i) country of origin, (ii) cacao cultivar, and (iii) fermentation method. The other three features are binary: (iv) use of starter culture, (v) turning of fermenting mass, and (vi) controlled temperature during fermentation. Experiments with features reported as unknown or missing were not considered for any PCA. Only the posterior samples contained within their 95% credible interval (CI) from each chain in the MCMC-NUTS runs were taken into account to perform PCA. The assessment of groups within the PCA results was realized only for features with more than one successful fit.

Moreover, PCAs were also performed over subgroups of parameter defined by the type of parameter and its association with a certain microbial group. Considered subgroups consisted of: (i) all MI parameters, (ii) maximum specific growth rates, (iii) mortality rates, (iv) yield coefficients, (v) Y-related parameters, (vi) LAB-related parameters, and (vii) AAB-related parameters. No PCA was run over substrate saturation constants due to their known correlation with maximum growth rates [47]. All PCAs used mean-centred data with no scaling, given that solutions of MIs were determined over scaled time series.

Finally, pairwise squared Mahalanobis distances (*D*_*M*_, [48]) were computed between grouping classes of each feature to quantify the magnitude of their separation. To achieve this, centroids of PCA scores from principal components 1 (PC1) and 2 (PC2) were computed for each *j* grouping class within an *i* feature and used to determine *D*_*M*_ as

2.15 $$D_{M}({\mathrm{PC}1}_{i,j},{\mathrm{PC}2}_{i,j})={\left({\bar{x}}_{1}-{\bar{x}}_{2}\right)}^{T}\;S^{-1}\;({\bar{x}}_{1}-{\bar{x}}_{2}),$$

where ${\bar{x}}_{1}$ and ${\bar{x}}_{2}$ are the centroid values of the scores of PC1_*i*,*j*_ and PC2_*i*,*j*_ respectively; and **S**^−1^ is the inverse of the covariance matrix between groups classes [48,49].

Both, PCA and *D*_*M*_ were implemented in R using functions *prcomp* [36] and *pairwise.mahalanobis* [50], respectively.

## 3. Results

### 3.1. First assessment of the models

First, we want to understand how well the different models—the baseline model from [9] and the MIs containing one or more of the additional mechanisms—perform. In order to identify differences in the success rate of the model variants, we apply every MI to every fermentation dataset. Table 4 summarizes the result.

**Table 4.** Summary of successful fits across 31 models iterations (MIs) and baseline.

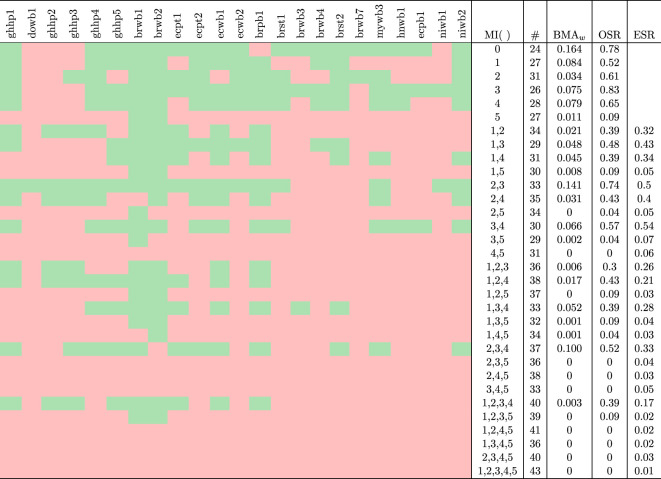

Light green-coloured cells indicate successful fits. Light-red coloured cells indicate non-successful fits. Columns ‘MI( )’, ‘#’, ‘BMA_*w*_’, ‘OSR’ and ‘ESR’ refer to combination of mechanisms deployed in model iteration, number of parameters, averaged Bayesian model averaging weights, observed success rate and expected success rate, respectively.

**Figure 2.**
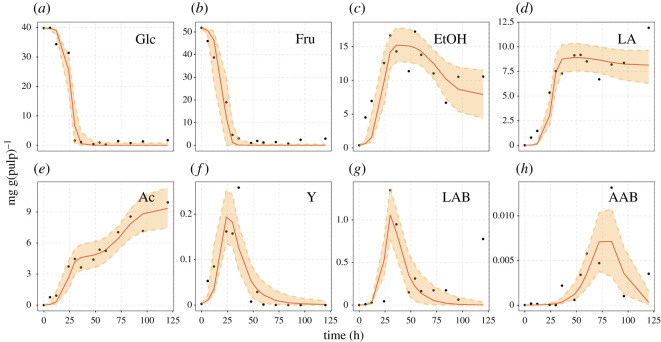
Posterior predictions of model iteration (MI) corresponding to mechanisms M2 and M3, MI(2,3), fitted to dataset *mywb3* reported by Papalexandratou *et al.* [19]. Metabolites: (*a*) glucose, (*b*) fructose, (*c*) ethanol, (*d*) lactic acid and (*e*) acetic acid. Microbial groups: (*f*) yeast, (*g*) lactic acid bacteria, and (*h*) acetic acid bacteria. Solid red lines represent posterior medians of the posterior predictions, solid black points denote experimental data and orange ribbon describe the 95% credible interval of posterior predictions.

In general terms, MIs summed up to 1024 runs over 32 available datasets; of which, 207 resulted in successful fits with values of $\hat{R}$ below 1.05, bulk-ESS and tail-ESS higher than 100 indicating that convergence of the MCMC-NUTS was accomplished (see electronic supplementary material, tables S3–S5). Furthermore, in terms of autocorrelation, all successful fits showed averaged values of ESS/N_draws_ over 12.5% (see electronic supplementary material, table S6), indicating an acceptable ESS above 1000. A number of nine datasets reported by Lefeber *et al.* [51,52], Moreira *et al.* [18], Bastos *et al.* [53] and Racine *et al.* [54] were not possible to fit with any MI at all. The remaining 23 fermentation datasets constitute the scope of our further investigation (table 1). As an example, figure 2 shows one MI, MI(2,3) describing the time series of one of the datasets (*mywb3* from Papalexandratou *et al.* [19]).

A striking observation is that the vast majority of MIs involving M5 were not able to produce successful fits to experimental data. Among these, exceptions are datasets described by Papalexandratou *et al.* [14], *brwb1* and *brwb2*. Both were well fitted by MI(5), MI(1,5) and MI(1,2,3,5); while MI(2,5) and MI(3,5) fitted *brwb1* and MI(1,4,5) fitted *brwb2* only. Thus, MIs (4,5), (2,3,5), (2,4,5), (3,4,5), (1,2,4,5), (1,3,4,5), (2,3,4,5) and (1,2,3,4,5) could not describe any dataset at all (table 4).

### 3.2. Model success

Both approaches for assessing model success across all datasets, a formal one as BMA and our proposed measures OSR and ESR, showed similar conclusions over competing MIs.

From their computations, the baseline model (MI(0)) showed the highest values (BMA_*w*_ = 0.164, OSR = 0.78). For MIs containing single mechanisms, M(1), M(2), M(3), M(4) and M(5), BMA_*w*_ were 0.084, 0.034, 0.075, 0.079 and 0.011, while OSR values were 0.52, 0.61, 0.83, 0.65 and 0.09, respectively. Among more complex combinations of mechanisms, BMA_*w*_ ranged between 0.00 to a maximum of 0.141 reached by the combination of M2 and M3 (MI(2,3)). Similarly, among these, MI(2,3) had a maximum OSR of 0.74 as listed in table 4.

Note that in general we expect a decrease of OSR with an increasing number of parameters in the model, due to higher complexity. Values for the single-mechanism MIs are therefore not directly comparable to the one of the baseline model. For even larger MIs (composite mechanisms), we have the ESR to partially correct for this.

Pertaining to ESRs, leaving out non-successful MIs, 2 out of 18 MIs showed higher values than their corresponding OSRs. These two MIs with higher ESRs correspond to iterations including M5 (i.e. MI(2,5) and MI(3,5)). Leaving aside MI(2,5) and MI(3,5) due to be the only exceptions of M5 ending up in successful fits, in overall combinations of mechanisms seems to lead to increases of their OSR over ESR on describing different datasets despite not overpassing the OSR of the baseline model (table 4).

### 3.3. Posterior predictions

Next, we resort to the distributions of posterior probabilities, in order to assess differences in the quality of the fit for the different MIs. Among the 23 datasets that were fitted by at least one MI, posterior predictions describe their dynamics remarkably well. In each data collection, despite the presence of highly influential observations and sampling rates ranging from 6 to 17 data points, time courses are simulated to an acceptable level. Again we refer to the example shown in figure 2, showing the fit of MI(2,3) to the dataset *mywb3* (see electronic supplementary material, figures S2–S17 for posterior predictions made by MI(2,3) and electronic supplementary material, figures S18–S23 for MIs, where M(2,3) was not suitable).

In terms of predictive accuracy among MIs fitted on each dataset, there were no outstanding differences on the basis of obtained PSIS-LOO deviance values that had overlapping standard errors between each other. Thus, is not surprising that for these cases posterior predictions resulted to be extremely similar (see electronic supplementary material, figure S24 for an example). Nevertheless, slight distinct PSIS-LOO were observed towards favouring MIs involving M1 in for datasets *brpb1*, *brwb4*, *brst2* and *niwb2* (see electronic supplementary material, figure S1). The latter is also evident by higher BMA weights by such MIs (see electronic supplementary material, table S7).

These subtle differences provided visually better fits by MI(1) with respect to MI(2,3) for datasets *brpb1* (see electronic supplementary material, figure S25). However, the same does not seem to be clear for *niwb2* where predictions made by M(1) and M(2,3) overlay each other with no evident improvements for either MI (see electronic supplementary material, figure S26).

### 3.4. Fermentation features

We now turn to the second question raised, namely whether the model parameters obtained by describing the datasets with all MIs are informative of the fermentation features behind the datasets. Via principal component analysis performed on the full parameter vectors or biologically meaningful subsets of the parameters, we want to assess whether distinct clusters emerge in agreement with differences in fermentation set-ups.

After dropping the use of a starter culture as a feature (see electronic supplementary material, S2), a total of 490 PCAs were performed from the remaining five features and seven parameters subsets. Note that MI(1,4) did not converge for datasets representing more than one used fermentation method, and MI(1), MI(1,2), MI(1,3), MI(1,4), MI(2,4), MI(1,2,3), MI(1,2,4), MI(1,3,4), MI(2,3,4) and MI(1,2,3,4) were not capable of describing datasets with more than one class of controlled temperature (see electronic supplementary material, figure S27).

In terms of group separation measured by *D*_*M*_ for cases with more than one pairwise comparison, medians of all *D*_*M*_ were computed (${\tilde{D}}_{M}$) as a way to visualize the magnitude of separation as single values. From this analysis, it can be described in general terms that cultivar, temperature and fermentation method showed the highest ${\tilde{D}}_{M}$ values; while, origin countries and turning of fermenting mass showed almost no separation between groups (with the exception of a few cases). Details are provided in the following subsections (see also electronic supplementary material, figure S27).

#### 3.4.1. Grouping of fermentation trials according to cultivar

PCAs with cultivar as feature of interest showed a consistent pattern of high values for ${\tilde{D}}_{M}$ with special emphasis on the subgroup of all parameters. With regard to MIs, clearer separations were the product of mostly complex MIs involving combinations of M2, M3 and M4 (see electronic supplementary material, figure S27, panel (*b*)).

**Figure 3.**
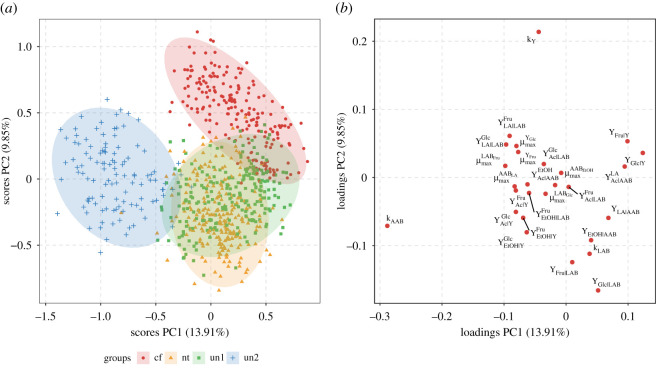
PCA score (*a*) and loading plot (*b*) from all parameters of model iteration MI(2,3), feature cultivar. For visualization purposes, scores of only 10% of posterior draws are shown. Criollo/Forastero (cf), Nacional/Trinitario (nt), unknown cultivar used by Camu *et al.* [13] (un1) and unknown cultivar used by Pereira *et al.* [16] (un2) are shown. Parameters located on the left and right with respect to 0 in PC1 loading plot determine differentiation between cf, nt and un2.

Figure 3 shows a PCA plot for MI(2,3). Among the four cultivar varieties, three showed a clear separation, namely Criollo/Forastero, Nacional/Trinitario and *un2* with explained variances of 13.91% by the first PCA component (PC1) and 9.85% by the second component (PC2). From its loading plot (figure 3*b*), parameters with negative loadings in PC1, mainly $\mu_{\max}^{Y_{\mathrm{Glc}}}$, $\mu_{\max}^{Y_{\mathrm{Fru}}}$, $\mu_{\max}^{{\mathrm{LAB}}_{\mathrm{Glc}}}$, $\mu_{\max}^{{\mathrm{LAB}}_{\mathrm{Fru}}}$, $\mu_{\max}^{{\mathrm{AAB}}_{\mathrm{LA}}}$, $\mu_{\max}^{{\mathrm{AAB}}_{\mathrm{EtOH}}}$, $Y_{\mathrm{LA}|\mathrm{LAB}}^{\mathrm{Glc}}$, $Y_{\mathrm{LA}|\mathrm{LAB}}^{\mathrm{Fru}}$, $Y_{\mathrm{EtOH}|\mathrm{LAB}}^{\mathrm{Glc}}$, $Y_{\mathrm{EtOH}|\mathrm{LAB}}^{\mathrm{Fru}}$, $Y_{\mathrm{EtOH}|Y}^{\mathrm{Glc}}$, $Y_{\mathrm{EtOH}|Y}^{\mathrm{Fru}}$, $Y_{\mathrm{Ac}|\mathrm{LAB}}^{\mathrm{Glc}}$, $Y_{\mathrm{Ac}|\mathrm{AAB}}^{\mathrm{EtOH}}$, $Y_{\mathrm{Ac}|Y}^{\mathrm{Glc}}$, $Y_{\mathrm{Ac}|Y}^{\mathrm{Fru}}$, *k*_Y_ and *k*_AAB_ determine the classes separation.

#### 3.4.2. Grouping of fermentation trials according to temperature control

**Figure 4.**
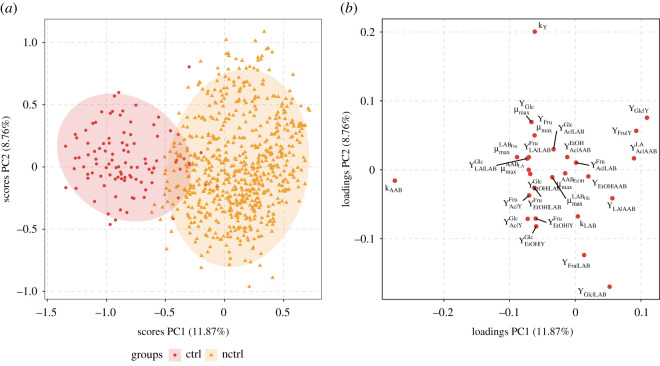
PCA score (*a*) and loading plot (*b*) from all parameters of model iteration MI(2,3), feature temperature. For visualization purposes, scores of only 10% of posterior draws are shown. Controlled temperature (ctrl) and non-controlled temperature (nctrl) are shown. Parameters located on the left and right with respect to 0 in PC1 loading plot determine differentiation between ctrl and nctrl.

Temperature control showed its highest ${\tilde{D}}_{M}$ values with MIs involving M2 (see electronic supplementary material, figure S27, panel (*e*)). The PCA of the set of all parameters for MI(2,3) resulted in a clear separation of groups determined by the use of controlled and non-controlled temperature with PC1 and PC2 scores explaining 11.87% and 8.76% of variance, respectively (figure 4). Likewise, the same set of parameters as in §3.4.1, showed negative loadings in PC1, indicating that these are defining the observed separation.

#### 3.4.3. Grouping of fermentation trials according to method

Fermentation method as classification feature resulted in less clear separation patterns. Highest values of ${\tilde{D}}_{M}$ were observed for MI(2) (see electronic supplementary material, figure S27, panel (*c*)). In this case, a clear separation between fermentations carried out in stainless-steel tanks and the rest of fermentation methods can be seen with total explained variance of 16.61% by PC1 and 9.97% by PC2 (see electronic supplementary material, figure S28). Furthermore, parameters with positive loadings in PC1, namely $\mu_{\max}^{Y_{\mathrm{Glc}}}$, $\mu_{\max}^{Y_{\mathrm{Fru}}}$, $\mu_{\max}^{{\mathrm{LAB}}_{\mathrm{Glc}}}$, $\mu_{\max}^{{\mathrm{LAB}}_{\mathrm{Fru}}}$, $\mu_{\max}^{{\mathrm{AAB}}_{\mathrm{LA}}}$, $\mu_{\max}^{{\mathrm{AAB}}_{\mathrm{EtOH}}}$, $Y_{\mathrm{LA}|\mathrm{LAB}}^{\mathrm{Glc}}$, $Y_{\mathrm{LA}|\mathrm{LAB}}^{\mathrm{Fru}}$, $Y_{\mathrm{EtOH}|Y}^{\mathrm{Glc}}$, $Y_{\mathrm{EtOH}|Y}^{\mathrm{Fru}}$, $Y_{\mathrm{EtOH}|\mathrm{LAB}}^{\mathrm{Glc}}$, $Y_{\mathrm{EtOH}|\mathrm{LAB}}^{\mathrm{Fru}}$, $Y_{\mathrm{Ac}|\mathrm{AAB}}^{\mathrm{EtOH}}$ and *k*_AAB_ seem to influence the separation between stainless-steel tank and the rest of methods (see electronic supplementary material, figure S28, panel (*b*)).

#### 3.4.4. Grouping of fermentation trials according to country of origin and turning of fermenting mass

In contrast to previous features, country of origin and turning of fermenting mass have lower ${\tilde{D}}_{M}$ values for single mechanisms and less cases of notoriously high distances. Instead, combinations of mechanisms and other subgroups of parameters, rather than the whole set, led to better classes separations. For country of origin, PCAs over LAB-related parameters on MIs involving combinations of M1, M2 and M4 resulted in higher ${\tilde{D}}_{M}$. For turning of fermenting mass, there were no sizeable differences (see electronic supplementary material, figure S27, panels (*a*) and (*d*)). For this case PCA from MI(1,3,4) explains a total variance of 45.8% for PC1 and 25.89% for PC2. Furthermore, a clear separation between Brazil with respect to Ecuador and Ghana, is defined over PC1. From its loadings, parameters with positive loadings in PC1 ($\mu_{\max}^{{\mathrm{LAB}}_{\mathrm{Glc}}}$, *Y*_LA|LAB_ and *k*_LAB_) are clearly denoting the separation between classes (see electronic supplementary material, figure S29). Lastly, turning of fermenting mass did not show any separation either for MI nor any subset of parameters.

## 4. Discussion

### 4.1. Model plausibility

We have assessed a series of mathematical model variants (or MIs) for cocoa bean fermentation in terms of two levels of plausibility: success of each run of a MI over a given dataset, and success of each MI to adequately describe the whole range of fermentation datasets. In this sense, failure in fitting a certain MI might be a consequence of practical non-identifiability of the parameters caused by weakly informative observations or priors, misspecification of the model [42], and even due to known limitations of MCMC-NUTS, such as poor chain mixing in presence of multimodal posteriors [55] and its higher sensitivity to model parametrization with respect to other samplers [56]. Success of a model, quantitatively represented as OSR per MI, highly depends on its capability to describe as many datasets as possible. Correspondingly, fitting failure becomes an indirect diagnostic tool that serve us to argue whether hypothesized regulatory interactions of the cocoa bean fermentation process are actually likely to be an influencing factor in the observed fermentation time series.

In general, the mechanisms discussed here have shown in most cases that their stand-alone and concomitant inclusion in the baseline model lead to convincing OSRs values, in particular for M2, M3 and M4. We can consider a lack of success in some runs involving these mechanisms is the product of numerically conflicting combinations of mechanisms rather than possible misspecification of the whole MI itself.

On the other hand, wide fitting failure patterns in runs involving M5 (table 4) and to a lesser extent also for M1, leads to the conclusion that these two mechanisms are not such significant influencing factors in the fermentation data studied here.

In the following, we will elaborate on the implications of fitting failure and plausibility of each mechanism considered in descending order relative to their OSR of the stand-alone inclusion in the baseline model.

Starting with M3, production of Ac by Y’s metabolism has not been hypothesized from direct experimental measurements as is the case for other mechanisms. Instead, indirect kinetic studies of isolated strains have shown such an ability of some species of Y [16]. This property has also been argued to be a possible explanation of high Ac production yields where populations of AAB seemed incapable of producing such amounts, as proposed by Pereira *et al.* [17]. Hence, given the OSRs obtained from its inclusion in the series of MIs presented here, M3 can be considered as quantitative evidence backing up this role of Y during fermentation. We strongly believe that cases where these MIs did not succeed are the consequence of weakly informative priors incapable of being sampled properly.

With respect to M2, in light of the recent characterization of fructophilic lactic acid bacteria (FLAB) in cocoa bean fermentation processes [28], the level of OSR achievement of MIs involving M2 is not surprising. The existence of this bacterial group can then explain an apparent discrepancy in the amounts of Glc and Fru consumed during the process. Despite both substrates being depleted in parallel, Glc is usually consumed first as Y populations reach their end. Thus, uptake of remaining Fru might be a consequence of FLAB activity. In contrast to M3, fitting failure of M2 might be a result of weakly informative observations in some of the datasets with no time lag between Glc and Fru consumption.

Similarly to M3, the reasoning behind M4 relies on indirect characterization of Y strains capable of metabolizing LA into EtOH [12] via their known metabolic pathways producing pyruvate from LA [29,30] for further production of EtOH [4]. However, in contrast to M3, M4 obtained an appreciable OSR for its stand-alone iteration rather than for its occurrence jointly with either M2 and M3. As an example, consider MI(2,3) compared with MI(2,4) and MI(3,4). While MI(2,3) stands out as the MI with more than one mechanism with largest OSR (equal to 0.74), its counterparts involving M4 perform poorly with OSRs equal to 0.43 for MI(2,4) and 0.57 for MI(3,4). In our opinion, this counterintuitive performance of M4 when combined with M2 and M3 can be the result of numerical issues due to conflicting interactions of these mechanisms preventing the Bayesian optimization to sample from complicated geometries of the posterior target, rather than biological causes against M4.

MIs employing M1 and M5 resulted in the lowest OSRs among all, both stand-alone and combined with other mechanisms. However, important distinctions need to be made between these two. First, M1 is formulated on the basis of several experimental studies that have brought evidence of metabolites diffusing into the bean [11,13,14,16,17], e.g. EtOH, LA and Ac, besides evaporation and degradation processes not directly measured, but highly likely. In this sense, low success of MIs accounting M1 can be due to a lack of the models to describe dynamics of metabolites diffusing into the inner bean. In particular, the pure degradation mechanism included in our MI does not fully account for all possible sinks (e.g. due to diffusion and evaporation) of these substances. Cases in which M1 and its iterations resulted in successful fits, are those where clear decreases of EtOH, LA and Ac are visible in the time series (see electronic supplementary material, figures S18, S19 and S25).

Second, regardless of the widely accepted mechanism of AAB consuming Ac once EtOH concentration has reached minimum levels in the fermenting mass [3,4,6,31], we have found solid quantitative evidence through assessing M5 that such a phenomenon has a very small impact on the process dynamics and thus is unlikely. By the end of fermentation, the AAB populations have been highly diminished and, in most of the datasets considered here, drops in Ac concentration were seldom reported. In other words, if M5 actually has an impact on the whole process, it would be necessary that AAB counts remain viable up to its completion in order to deplete Ac. This observation can also explain the few exceptions, in which M5 led to successful fits. In total, the vast majority of datasets showed minimal counts of AAB even before the penultimate day of fermentation, with the exception of *brwb1* and *brwb2* reported by Papalexandratou *et al.* [14], where drops of AAB counts are quite abrupt by its last day, limiting the capability of these MIs to simulate a complete diminution of its population (see electronic supplementary material, figures S8 and S9).

Finally, after reviewing all above-mentioned causes of fitting failure and how they could have affected success ratios of each MI, it is plausible to assume that harsher combinations of such causes are responsible for failing to fit any model to nine datasets (see §3.1) as well for some others that were scarcely described, e.g. *dowb1* and *niwb2*.

### 4.2. Interpretation of the posterior distributions of model parameters

Here, we would like to discuss general aspects of the posterior distributions and how they allow us to further assess the different MIs. We will focus on the agreement of the obtained posteriors with values in the literature as well as practical non-identifiability of parameters.

For parameter agreement, let us consider the set of posterior distributions for MI(2,3) fitted over several datasets (see electronic supplementary material, table S8). As stated in [9], among all estimated posteriors, few did not agree with reported estimates in the literature. Those which culminated in values far away from reported ranges (more than 10-fold) as well as others, whose biological plausibility is unlikely, suggest evidence of practical non-identifiability, as evidenced by posteriors with wide credible intervals. This might be due to weakly informative observations that do not capture entirely the dynamics of the included mechanisms [9,42].

This assumption seems to be supported by parameters' posteriors under the scope of different MIs. By the inclusion of extra terms acting upon the dynamics, in which non-identifiable parameters are suspected to exist, their wide ranges should be visibly reduced. In fact, focusing on examples already mentioned in [9] (particularly *Y*_LA|AAB_, *Y*_EtOH|AAB_ and $Y_{\mathrm{Ac}|\mathrm{AAB}}^{\mathrm{LA}}$), we can see this reduction across different MIs describing datasets *ghhp1*, *ecwb1*, *ecwb2* and *brpb1* (see electronic supplementary material, figure S30).

Nevertheless, inclusion of extra parameters does not entirely eliminate non-identifiability, which suggests the need for more informative priors for further developments of cocoa bean fermentation modelling.

### 4.3. Grouping of fermentation features

We have seen that fermentation features can be distinguishable with respect to all parameter estimates derived from their posterior distributions, especially those from MI(2) and MI(2,3). From our perspective, this is a clear illustration, how ODE-based modelling, rather than the usual methods of chemical fingerprinting [57–59], can demonstrate differences in features of the process.

An example is the clear differences in fermentation features identified through kinetic parameter estimates of MI(2,3) for cultivar, controlled temperature and fermentation methodology. Regarding cultivar, similar findings have been reported for biochemical characterization studies where the same cultivars used in different countries have shown to be part of similar classes within PCA [57]. This would then explain why country of origin used as a feature, did not result in clear separation patterns. Instead, only few subsets of parameters for certain MIs ended up in clear group separations in that case, as it happens for the LAB-related parameters in MI(1,3,4) (see §3.4.4). This could be an indicator that indeed dynamics of different LAB populations are linked to the location where fermentation took place [5].

In a similar fashion, grouping of fermentation trials dependent on whether they were performed under controlled temperature settings, reflects how kinetic parameters of MI(2,3) might be influenced by this feature. In this regard, explicit inclusion of temperature in these models would be a natural option. From our point of view, we firmly consider that incorporation of temperature in this modelling scheme would be beneficial in case remarkable improvements on the assessing statistics presented here were seen. However, explicitly incorporating temperature as a dynamical variable did not dramatically change either PSIS-LOO, posterior predictions or parameter ranges towards more biological plausibility (see electronic supplementary material, S5).

Furthermore, classification of trials with respect to methodology also tends to lead to a clear separation with a special emphasis on trials performed in stainless-steel tanks fitted with MI(2). This finding suggests that the use of stainless-steel tanks affects kinetic parameters, making them distinct from other methods. Besides, it becomes an indication that inclusion of M2 seems to be driving this difference, as well as other feature discriminations. The latter observation is based on the loading plots that for all these PCAs are determined by parameters related to M2, such as $\mu_{\max}^{{\mathrm{LAB}}_{\mathrm{Fru}}}$, $Y_{\mathrm{EtOH}|\mathrm{LAB}}^{\mathrm{Fru}}$ and $Y_{\mathrm{LA}|\mathrm{LAB}}^{\mathrm{Fru}}$.

Lastly, it is worth remarking that including all parameters’ posterior draws contained in their 95% CI for conducting PCAs plays an important role in finding more meaningful differences between the features. This conclusion comes from a former approach where we performed PCAs over the medians of each chain of successful MIs instead. In such an exercise, besides obvious quantitative differences between its PCs and their counterparts reported here (see electronic supplementary material, table S9), the same classification of the features was observed, with the exception of a noticeable separation between all countries (see electronic supplementary material, figure S31) rather than the overlap between Ghana and Ecuador shown in electronic supplementary material, figure S29, which could have been mistakenly ignored if it were not by considering the posterior distributions’ uncertainties.

## 5. Conclusion

The series of MIs presented here constitute a first kinetic exploration of the plausibility of regulatory dynamics of cocoa bean fermentation not considered in our previous modelling [9], but long reported and hypothesized in the literature. Thus, it allows us to evaluate the plausibility of various mechanisms in a stand-alone and concomitant manner. Among the five mechanisms discussed here, M2 (consumption of fructose by lactic acid bacteria) and M3 (production of acetic acid by yeast) have gathered the strongest support in our investigation.

Our scheme also allows us to conclude that loss of metabolites by physical phenomena (M1) is quite minimal, relative to their consumption and formation rates emphasizing the importance of microbial biochemical processes. Furthermore, it also offers quantitative evidence that a widely hypothesized mechanism, M5 (over-oxidation of acetic acid by acetic acid bacteria), does not agree with experimental data.

With reference to fermentation features, the rich set of parameter estimation results grants for interpretation on three levels: (i) We find that the parametrized time courses separate different fermentation features with different quality. Across all models, origin countries seem to only have a small influence on systematic time course differences. By contrast, temperature and cultivar seem to have a strong effect on fermentation dynamics (and hence on systematic differences in the resulting parameter vectors). (ii) Orthogonal to this view, we can assess which model versions lead to better discrimination fermentation features compared with the basic fermentation model from [9]. This complements our assessment of parameter estimation success, which is summarized in table 4. (iii) By splitting parameters into groups, we can assess the involvement of certain microorganisms in the systematic differences between fermentation features.

Lastly, this work commends that in a pure sense of describing fermentation dynamics in the pulp, inclusion of temperature as a dynamical variable does not add improvements to fits obtained under the proposed scheme. However, future advancements in cocoa bean fermentation modelling might find it necessary to take it into account for a more refined description of more detailed experimental data and to capture its reported importance in mediating important dynamics, for instance, its role in diffusion processes of acids into the bean [60].

## Data accessibility

Data and relevant code for this research work are stored in GitHub https://github.com/mmorenozam/ecbfm and have been archived within the Zenodo Repository https://zenodo.org/record/5510370 [61]. Supplementary results are provided in electronic supplementary material [62].

## References

[1] Lopez AS, Dimick PS 1995 Cocoa fermentation. In *Biotechnology*, 2nd edn, vol. 9 (eds G Reed, TW Nagodawithana), ch. 14, pp. 561–577. Weinheim, Germany: VCH Verlagsgesellschaft mbH.

[2] Schwan RF, Pereira GV d. M, Fleet GH. 2014 Microbial activities during cocoa fermentation. In *Cocoa and coffee fermentations* (eds RF Schwan, GH Fleet), ch. 4, pp. 129–192. Boca Raton, FL: CRC Press.

[3] De Vuyst L, Weckx S. 2016 The cocoa bean fermentation process: from ecosystem analysis to starter culture development. J. Appl. Microbiol. **121**, 5-17. (10.1111/jam.13045) 26743883

[4] De Vuyst L, Leroy F. 2020 Functional role of yeasts, lactic acid bacteria, and acetic acid bacteria in cocoa fermentation processes. FEMS Microbiol. Rev. **44**, 432-453. (10.1093/femsre/fuaa014) 32420601

[5] Figueroa-Hernández C, Mota-Gutierrez J, Ferrocino I, Hernández-Estrada ZJ, González-Ríos O, Cocolin L, Suárez-Quiroz ML. 2019 The challenges and perspectives of the selection of starter cultures for fermented cocoa beans. Int. J. Food Microbiol. **301**, 41-50. (10.1016/j.ijfoodmicro.2019.05.002) 31085407

[6] Schwan RF, Wheals AE. 2004 The microbiology of cocoa fermentation and its role in chocolate quality. Crit. Rev. Food Sci. Nutr. **44**, 205-221. (10.1080/10408690490464104) 15462126

[7] Kresnowati MTAP, Gunawan AY, Muliyadini W. 2015 Kinetics model development of cocoa bean fermentation. AIP Conf. Proc. **1699**, 030004. (10.1063/1.4938289)

[8] López-Pérez PA, Cuervo-Parra JA, Robles-Olvera VJ, Jimenes GDCR, España VHP, Romero-Cortes T. 2018 Development of a novel kinetic model for cocoa fermentation applying the evolutionary optimization approach. Int. J. Food Eng. **14**, 20170206. (10.1515/ijfe-2017-0206)

[9] Moreno-Zambrano M, Grimbs S, Ullrich MS, Hütt M-T. 2018 A mathematical model of cocoa bean fermentation. R. Soc. Open Sci. **5**, 180964. (10.1098/rsos.180964) 30473841 PMC6227950

[10] John WA, Böttcher NL, Behrends B, Corno M, D’souza RN, Kuhnert N, Ullrich MS. 2020 Experimentally modelling cocoa bean fermentation reveals key factors and their influences. Food Chem. **302**, 125335. (10.1016/j.foodchem.2019.125335) 31416001

[11] Camu N, De Winter T, Verbrugghe K, Cleenwerck I, Vandamme P, Takrama JS, Vancanneyt M, De Vuyst L. 2007 Dynamics and biodiversity of populations of lactic acid bacteria and acetic acid bacteria involved in spontaneous heap fermentation of cocoa beans in Ghana. Appl. Environ. Microbiol. **73**, 1809-1824. (10.1128/AEM.02189-06) 17277227 PMC1828797

[12] Lagunes Gálvez S, Loiseau G, Paredes JL, Barel M, Guiraud J-P. 2007 Study on the microflora and biochemistry of cocoa fermentation in the Dominican Republic. Int. J. Food Microbiol. **114**, 124-130. (10.1016/j.ijfoodmicro.2006.10.041) 17187887

[13] Camu N, Van Schoor A, De Bruyne K, Vandamme P, Takrama JS, Addo SK, De Vuyst L. 2008 Influence of turning and environmental contamination on the dynamics of populations of lactic acid and acetic acid bacteria involved in spontaneous cocoa bean heap fermentation in Ghana. Appl. Environ. Microbiol. **74**, 86-98. (10.1128/AEM.01512-07) 17993565 PMC2223199

[14] Papalexandratou Z, Vrancken G, Bruyne KD, Vandamme P, Vuyst LD. 2011 Spontaneous organic cocoa bean box fermentations in Brazil are characterized by a restricted species diversity of lactic acid bacteria and acetic acid bacteria. Food Microbiol. **28**, 1326-1338. (10.1016/j.fm.2011.06.003) 21839382

[15] Papalexandratou Z, Falony G, Romanens E, Jimenez JC, Amores F, Daniel H-M, De Vuyst L. 2011 Species diversity, community dynamics, and metabolite kinetics of the microbiota associated with traditional Ecuadorian spontaneous cocoa bean fermentations. Appl. Environ. Microbiol. **77**, 7698-7714. (10.1128/AEM.05523-11) 21926224 PMC3209185

[16] Pereira GVdM, Miguel MGdCP, Ramos CL, Schwan RF. 2012 Microbiological and physicochemical characterization of small-scale cocoa fermentations and screening of yeast and bacterial strains to develop a defined starter culture. Appl. Environ. Microbiol. **78**, 5395-5405. (10.1128/AEM.01144-12) 22636007 PMC3416416

[17] Pereira GVdM, Teixeira Magalhães K, de Almeida EG, da Silva Coelho I, Schwan RF. 2013 Spontaneous cocoa bean fermentation carried out in a novel-design stainless steel tank: influence on the dynamics of microbial populations and physical–chemical properties. Int. J. Food Microbiol. **161**, 121-133. (10.1016/j.ijfoodmicro.2012.11.018) 23279821

[18] Moreira IMdV, Miguel MGdCP, Duarte WF, Dias DR, Schwan RF. 2013 Microbial succession and the dynamics of metabolites and sugars during the fermentation of three different cocoa (*Theobroma cacao* L.) hybrids. Food Res. Int. **54**, 9-17. (10.1016/j.foodres.2013.06.001)

[19] Papalexandratou Z, Lefeber T, Bahrim B, Lee OS, Daniel H-M, De Vuyst L. 2013 *Hanseniaspora opuntiae*, *Saccharomyces cerevisiae*, *Lactobacillus fermentum*, and *Acetobacter pasteurianus* predominate during well-performed Malaysian cocoa bean box fermentations, underlining the importance of these microbial species for a successful cocoa bean fermentation process. Food Microbiol. **35**, 73-85. (10.1016/j.fm.2013.02.015) 23664257

[20] Romanens E, Näf R, Lobmaier T, Pedan V, Leischtfeld SF, Meile L, Schwenninger SM. 2018 A lab-scale model system for cocoa bean fermentation. Appl. Microbiol. Biotechnol. **102**, 3349-3362. (10.1007/s00253-018-8835-6) 29492640

[21] Lee AH et al. 2019 A laboratory-scale model cocoa fermentation using dried, unfermented beans and artificial pulp can simulate the microbial and chemical changes of on-farm cocoa fermentation. Eur. Food Res. Technol. **245**, 511-519. (10.1007/s00217-018-3171-8)

[22] Papalexandratou Z et al. 2019 Linking cocoa varietals and microbial diversity of Nicaraguan fine cocoa bean fermentations and their impact on final cocoa quality appreciation. Int. J. Food Microbiol. **304**, 106-118. (10.1016/j.ijfoodmicro.2019.05.012) 31176963

[23] Monod J. 1949 The growth of bacterial cultures. Annu. Rev. Microbiol. **3**, 371-394. (10.1146/annurev.mi.03.100149.002103)

[24] Contois DE. 1959 Kinetics of bacterial growth: relationship between population density and specific growth rate of continuous cultures. Microbiology **21**, 40-50. (10.1099/00221287-21-1-40) 13811643

[25] Watson HE. 1908 A note on the variation of the rate of disinfection with change in the concentration of the disinfectant. J. Hyg. **8**, 536-542. (10.1017/S0022172400015928) PMC2167149 20474372

[26] De Vuyst L, Lefeber T, Papalexandratou Z, Camu N. 2010 The functional role of lactic acid bacteria in cocoa bean fermentation. In *Biotechnology of lactic acid bacteria*, ch. 17, pp. 301–325. Ames, IA: John Wiley & Sons, Ltd. (10.1002/9780813820866.ch17) 22365351

[27] Lefeber T, Janssens M, Camu N, De Vuyst L. 2010 Kinetic analysis of strains of lactic acid bacteria and acetic acid bacteria in cocoa pulp simulation media toward development of a starter culture for cocoa bean fermentation. Appl. Environ. Microbiol. **76**, 7708-7716. (10.1128/AEM.01206-10) 20889778 PMC2988588

[28] Viesser JA, de Melo Pereira GV, de Carvalho Neto DP, Azevedo V, Brenig B, Rogez H, Góes-Neto A, Soccol CR. 2020 Exploring the contribution of fructophilic lactic acid bacteria to cocoa beans fermentation: isolation, selection and evaluation. Food Res. Int. **136**, 109478. (10.1016/j.foodres.2020.109478) 32846561

[29] Moens F, Lefeber T, De Vuyst L. 2014 Oxidation of metabolites highlights the microbial interactions and role of *Acetobacter pasteurianus* during cocoa bean fermentation. Appl. Environ. Microbiol. **80**, 1848-1857. (10.1128/AEM.03344-13) 24413595 PMC3957632

[30] Papalexandratou Z, Camu N, Falony G, De Vuyst L. 2011 Comparison of the bacterial species diversity of spontaneous cocoa bean fermentations carried out at selected farms in Ivory Coast and Brazil. Food Microbiol. **28**, 964-973. (10.1016/j.fm.2011.01.010) 21569940

[31] Crafack M et al. 2013 Influencing cocoa flavour using *Pichia kluyveri* and *Kluyveromyces marxianus* in a defined mixed starter culture for cocoa fermentation. Int. J. Food Microbiol. **167**, 103-116. (10.1016/j.ijfoodmicro.2013.06.024) 23866910

[32] Gabry J, Simpson D, Vehtari A, Betancourt M, Gelman A. 2019 Visualization in Bayesian workflow. J. R. Stat. Soc. A (Stat. Soc.) **182**, 389-402. (10.1111/rssa.12378)

[33] Gelman A, Hwang J, Vehtari A. 2014 Understanding predictive information criteria for Bayesian models. Stat. Comput. **24**, 997-1016. (10.1007/s11222-013-9416-2)

[34] Strachan RW, van Dijk HK. 2003 Bayesian model selection with an uninformative prior. Oxford Bull. Econom. Stat. **65**, 863-876. (10.1046/j.0305-9049.2003.00095.x)

[35] Carpenter B et al. 2017 Stan: a probabilistic programming language. J. Stat. Softw. **76**, 1-32. (10.18637/jss.v076.i01) PMC9788645 36568334

[36] R Core Team. 2020 *R: A language and environment for statistical computing*. Vienna, Austria: R Foundation for Statistical Computing. See https://www.R-project.org/.

[37] Stan Development Team. 2020 RStan: the R interface to Stan. R package version 2.21.1.

[38] Hoffman MD, Gelman A. 2014 The no-u-turn sampler: adaptively setting path lengths in Hamiltonian Monte Carlo. J. Mach. Learn. Res. **15**, 1593-1623.

[39] Dormand J, Prince P. 1980 A family of embedded Runge-Kutta formulae. J. Comput. Appl. Math. **6**, 19-26. (10.1016/0771-050X(80)90013-3)

[40] Ahnert K, Mulansky M. 2011 Odeint – solving ordinary differential equations in C++. AIP Conf. Proc. **1389**, 1586-1589. (10.1063/1.3637934)

[41] Vehtari A, Gelman A, Simpson D, Carpenter B, Bürkner P-C. 2021 Rank-normalization, folding, and localization: an improved $\hat{R}$ for assessing convergence of MCMC (with discussion). Bayesian Anal. **16**, 667-718. (10.1214/20-BA1221)

[42] Gelman A et al. 2020 Bayesian workflow. (http://arxiv.org/abs/2011.01808)

[43] Hoeting JA, Madigan D, Raftery AE, Volinsky CT. 1999 Bayesian model averaging: a tutorial. Stat. Sci. **14**, 382-417. (10.1214/ss/1009212519)

[44] Yao Y, Vehtari A, Simpson D, Gelman A. 2018 Using stacking to average Bayesian predictive distributions (with discussion). Bayesian Anal. **13**, 917-1007. (10.1214/17-BA1091)

[45] Dormann CF et al. 2018 Model averaging in ecology: a review of Bayesian, information-theoretic, and tactical approaches for predictive inference. Ecol. Monogr. **88**, 485-504. (10.1002/ecm.1309)

[46] Vehtari A, Gelman A, Gabry J. 2017 Practical Bayesian model evaluation using leave-one-out cross-validation and WAIC. Stat. Comput. **27**, 1413-1432. (10.1007/s11222-016-9696-4)

[47] Rosenbaum B, Raatz M, Weithoff G, Fussmann GF, Gaedke U. 2019 Estimating parameters from multiple time series of population dynamics using Bayesian inference. Front. Ecol. Evol. **6**, 234. (10.3389/fevo.2018.00234)

[48] Goodpaster AM, Kennedy MA. 2011 Quantification and statistical significance analysis of group separation in NMR-based metabonomics studies. Chemom. Intell. Lab. Syst. **109**, 162-170. (10.1016/j.chemolab.2011.08.009) PMC4523310 26246647

[49] Mahalanobis PC. 1936 On the generalised distance in statistics. In *Proc. National Institute of Sciences of India*, pp. 49–55.

[50] McFerrin L. 2013 HDMD: statistical analysis tools for high dimension molecular data (HDMD). R package version 1.2.

[51] Lefeber T, Gobert W, Vrancken G, Camu N, De Vuyst L. 2011 Dynamics and species diversity of communities of lactic acid bacteria and acetic acid bacteria during spontaneous cocoa bean fermentation in vessels. Food Microbiol. **28**, 457-464. (10.1016/j.fm.2010.10.010) 21356451

[52] Lefeber T, Papalexandratou Z, Gobert W, Camu N, De Vuyst L. 2012 On-farm implementation of a starter culture for improved cocoa bean fermentation and its influence on the flavour of chocolates produced thereof. Food Microbiol. **30**, 379-392. (10.1016/j.fm.2011.12.021) 22365351

[53] Bastos VS, Santos MF, Gomes LP, Leite AM, Flosi Paschoalin VM, Del Aguila EM. 2018 Analysis of the cocobiota and metabolites of *Moniliophthora perniciosa*-resistant *Theobroma cacao* beans during spontaneous fermentation in southern Brazil. J. Sci. Food Agric. **98**, 4963-4970. (10.1002/jsfa.9029) 29577311

[54] Racine KC, Lee AH, Wiersema BD, Huang H, Lambert JD, Stewart AC, Neilson AP. 2019 Development and characterization of a pilot-scale model cocoa fermentation system suitable for studying the impact of fermentation on putative bioactive compounds and bioactivity of cocoa. Foods **8**, 102. (10.3390/foods8030102) PMC6463099 30893898

[55] Mangoubi O, Pillai NS, Smith A. 2018 Does Hamiltonian Monte Carlo mix faster than a random walk on multimodal densities? (http://arxiv.org/abs/1808.03230)

[56] Monnahan CC, Thorson JT, Branch TA. 2017 Faster estimation of Bayesian models in ecology using Hamiltonian Monte Carlo. Methods Ecol. Evol. **8**, 339-348. (10.1111/2041-210X.12681)

[57] Caligiani A, Palla L, Acquotti D, Marseglia A, Palla G. 2014 Application of 1H NMR for the characterisation of cocoa beans of different geographical origins and fermentation levels. Food Chem. **157**, 94-99. (10.1016/j.foodchem.2014.01.116) 24679756

[58] Megías-Pérez R, Grimbs S, D’Souza RN, Bernaert H, Kuhnert N. 2018 Profiling, quantification and classification of cocoa beans based on chemometric analysis of carbohydrates using hydrophilic interaction liquid chromatography coupled to mass spectrometry. Food Chem. **258**, 284-294. (10.1016/j.foodchem.2018.03.026) 29655735

[59] Kumari N, Grimbs A, D’Souza RN, Verma SK, Corno M, Kuhnert N, Ullrich MS. 2018 Origin and varietal based proteomic and peptidomic fingerprinting of theobroma cacao in non-fermented and fermented cocoa beans. Food Res. Int. **111**, 137-147. (10.1016/j.foodres.2018.05.010) 30007670

[60] Biehl B, Passern D, Sagemann W. 1982 Effect of acetic acid on subcellular structures of cocoa bean cotyledons. J. Sci. Food Agric. **33**, 1101-1109. (10.1002/jsfa.2740331107)

[61] Moreno-Zambrano M, Ullrich MS, Hütt M-T. 2021 Data from: Exploring cocoa bean fermentation mechanisms by kinetic modelling. *Zenodo Digital Repository*. (10.5281/zenodo.5510370) PMC8847890 35223050

[62] Moreno-Zambrano M, Ullrich MS, Hütt M-T. 2022 Supplementary material from: Exploring cocoa bean fermentation mechanisms by kinetic modelling. *Figshare*. 10.1098/rsos.210274 PMC8847890 35223050

